# A Survey on Quantitative Risk Estimation Approaches for Secure and Usable User Authentication on Smartphones

**DOI:** 10.3390/s23062979

**Published:** 2023-03-09

**Authors:** Maria Papaioannou, Filippos Pelekoudas-Oikonomou, Georgios Mantas, Emmanouil Serrelis, Jonathan Rodriguez, Maria-Anna Fengou

**Affiliations:** 1 Instituto de Telecommunicaçoes, 3810-193 Aveiro, Portugal; 2 Faculty of Engineering and Science, University of Greenwich, Chatham Maritime, Kent ME4 4TB, UK; 3 Evotel Informática S.A., 27400 Lugo, Spain; 4 Netcompany-Intrasoft, 19002 Athens, Greece; 5 Faculty of Computing, Engineering and Science, University of South Wales, Pontypridd CF37 1DL, UK

**Keywords:** continuous user authentication, risk-based user authentication, quantitative risk estimation, mobile devices, security vs. usability

## Abstract

Mobile user authentication acts as the first line of defense, establishing confidence in the claimed identity of a mobile user, which it typically does as a precondition to allowing access to resources in a mobile device. NIST states that password schemes and/or biometrics comprise the most conventional user authentication mechanisms for mobile devices. Nevertheless, recent studies point out that nowadays password-based user authentication is imposing several limitations in terms of security and usability; thus, it is no longer considered secure and convenient for the mobile users. These limitations stress the need for the development and implementation of more secure and usable user authentication methods. Alternatively, biometric-based user authentication has gained attention as a promising solution for enhancing mobile security without sacrificing usability. This category encompasses methods that utilize human physical traits (physiological biometrics) or unconscious behaviors (behavioral biometrics). In particular, risk-based continuous user authentication, relying on behavioral biometrics, appears to have the potential to increase the reliability of authentication without sacrificing usability. In this context, we firstly present fundamentals on risk-based continuous user authentication, relying on behavioral biometrics on mobile devices. Additionally, we present an extensive overview of existing quantitative risk estimation approaches (QREA) found in the literature. We do so not only for risk-based user authentication on mobile devices, but also for other security applications such as user authentication in web/cloud services, intrusion detection systems, etc., that could be possibly adopted in risk-based continuous user authentication solutions for smartphones. The target of this study is to provide a foundation for organizing research efforts toward the design and development of proper quantitative risk estimation approaches for the development of risk-based continuous user authentication solutions for smartphones. The reviewed quantitative risk estimation approaches have been divided into the following five main categories: (i) probabilistic approaches, (ii) machine learning-based approaches, (iii) fuzzy logic models, (iv) non-graph-based models, and (v) Monte Carlo simulation models. Our main findings are summarized in the table in the end of the manuscript.

## 1. Introduction

The ubiquitous use of smartphones and mobile phones has become an indispensable aspect of our daily routine [1,2]. With an increasing number of people using smartphones every year, their ease of use and advanced performance capabilities enable users to perform more sensitive and critical tasks, making these devices into attractive and profitable targets for attackers. The authors in [1] provided a comprehensive survey on cybersecurity concerns in smartphones, while the authors in [2] presented a thorough classification of cybersecurity threats on mobile devices. In this context, it is important to highlight the importance of mobile user authentication which acts as the first line of defense, establishing confidence in the claimed identity of a mobile user, which it typically does as a precondition to allowing access to resources in a mobile device [3,4,5,6,7,8]. NIST [9] states that password schemes and/or biometrics comprise the most conventional user authentication mechanisms used in mobile devices. In particular, password-based user authentication methods (e.g., standard passwords, PINs, graphical passwords) have been used to verify the identity of a mobile user for several decades. Nevertheless, nowadays it is no longer considered secure or usable for the mobile user for various reasons [10,11,12]. For instance, Zhang at al. [13] have identified that mobile users struggle to memorize and accurately recall their passwords, especially when they are long enough to convey strong security. Consequently, users usually set passwords that are easy to recall, making their mobile devices vulnerable to various attacks such as dictionary, guessing, shoulder-surfing, smudge and keylogger-based attacks [13,14]. On top of that, Abuhamad et al. state that a major issue when implementing password-based user authentication is the underlying assumption of having equal security requirements for all applications [13,14].

On the other hand, biometric-based user authentication has gained attention as it appears to be able to enhance mobile authentication security without sacrificing usability, as behavioral modalities can be gathered and utilized, unobtrusively, for authentication purposes [11,12,15]. Biometric-based user authentication fall into two main categories: (i) physiological biometric-based authentication, which utilizes mobile users’ human physical characteristics, also referred to as physiological biometrics, such as fingerprints, facial traits, and hand geometries to authenticate a legitimate user; and (ii) behavioral biometric-based authentication, which makes use of mobile users’ involuntary actions, also referred to as behavioral biometrics, such as gait, and keystroke dynamics to validate their claimed identity [16]. In the last decade, popular mobile device manufacturers have already started embedding sensors, such as fingerprint readers, iris and face scanners, into their launched smartphones and deploying physiological biometrics for user authentication purposes. These are utilized as they appear to be secure since they are unique. Nevertheless, nowadays researchers have demonstrated that physiological biometric-based user authentication mechanisms can be hacked easily with inexpensive equipment and relatively primitive algorithms (e.g., using mobile users’ photos extracted from social media), making smartphones vulnerable to numerous attacks, including impersonation [17,18,19,20]. Alternatively, the behavioral biometrics cannot be shared, copied, lost or stolen, and thus behavioral biometric-based authentication mechanisms appear to be more secure and accurate [12]. On top of that, they authenticate users unobtrusively, based on their interactions with the device, and thus, when efficiently deployed, address the security versus usability challenge in mobile user authentication [3,4,5,6,7].

Furthremore, nowadays, continuous user authentication, relying on behavioral biometrics, has been shown to have the potential to further improve mobile authentication security without sacrificing usability (i.e., security and usability are often thought of as being contradictory) [12,21,22,23,24]. More specifically, continuous user authentication mechanisms have been proposed in the literature for use to continuously verify the user’s identity during their entire interaction with the smartphone device [25]. Specifically, the verification process of a user’s identity can be event-based, periodical (i.e., at fix intervals), or take place randomly. This allows us to overcome the limitations of the conventional one-shot authentication, where authentication happens only at the beginning of the session and, afterwards, any future changes and/or abnormalities in user identity/behavior remain undetected, increasing the risk of sensitive information leakage and user’s privacy violation [12,14,23].

Besides, it is worthwhile mentioning that enhancing continuous user authentication with the concept of risk-based authentication will lead to the following two benefits. The first one is that it increases the efficiency of continuous user authentication by only triggering the verification process for user re-authentication when it is actually required, minimizing the consumed resources. In particular, the verification process will be triggered based on real-time high-risk scores which are estimated only when an abnormality on a set of attributes has been detected (i.e., event-driven approach), instead of being triggered at fix intervals or randomly. On the other hand, the second benefit coming along with risk-based continuous authentication is the adaption of the user re-authentication levels autonomously on-the-fly based on the apparent risks [12,26]. For instance, during user’s session, when the real-time risk score is low (i.e., compared to a predefined threshold), the mobile user will remain signed in. Nevertheless, when the risk score is medium, uni-modal authentication (e.g., based on keystroke dynamics) will be required for re-authentication. Conversely, when the risk score is high, then multi-modal authentication (e.g., based on keystroke dynamics and voice) will be required for re-authentication to prove the claimed identity of the user and remain signed in or, otherwise, a countermeasure action will be taken (i.e., device lock) [12,27]. Therefore, it is clear that accurate risk estimation (i.e., computation of an accurate risk score of an action or event) plays a key role in risk-based continuous user authentication as it might impact its overall usability and security [27,28].

In principle, the different approaches which have been proposed over the years for risk estimation can be qualitative or quantitative [29]. Qualitative risk estimation approaches have been widely used in several areas until recently. However, despite appearing credible, existing qualitative approaches heavily rely on expert judgment and, therefore, the risk assessment is subjective, rendering these approaches inappropriate for use in real-world security solutions [30,31]. On the other hand, quantitative risk estimation approaches have emerged as a new tendency, addressing the subjective nature of qualitative risk estimation approaches, which is their major limitation, in order to improve their accuracy and reliability, which are two key characteristics for risk-based continuous user authentication on smartphones [32]. Nevertheless, so far, there has also been a lack of suitable quantitative risk estimation approaches for risk-based continuous user authentication for smartphones. Thus, more effort is required to investigate the suitability and limitations of these approaches for use in such mobile user authentication solutions. Therefore, this paper aims *to give an extensive overview of existing quantitative risk estimation approaches that could be possibly adopted in risk-based continuous user authentication solutions for smartphones in order to provide a foundation for organizing research efforts toward the design and development of proper quantitative risk estimation approaches to provide such user authentication solutions*. There is no doubt that selecting quality references is a crucial aspect of writing a survey paper. The methodology that we used to gather high-quality references to write this survey paper is as follows:

1. Before selecting references, we determined the scope of the survey paper, which is to investigate existing quantitative risk estimation approaches that could be possibly adopted in risk-based continuous user authentication solutions for smartphones.
2. Afterwards, we identified the most relevant databases and search engines, namely Google Scholar, IEEE Xplore, ACM Digital Library, and ScienceDirect.
3. Then, we used appropriate keywords to find relevant references (i.e., quantitative risk estimation, risk-based user authentication, continuous user authentication, behavioral biometrics), also utilizing Boolean operators (AND, OR, NOT) to refine our search. We also used advanced search options to further refine our search.
4. After that, we evaluated the quality of the references by: (i) looking for references that were published in reputable peer-reviewed journals or conference proceedings; (ii) checking the authors’ credentials and their affiliations; and (iii) looking for references that were recent and relevant to our topic.
5. Finally, we organized the references by creating a spreadsheet to keep track of all the references. Then, we analyzed each reference and identified the key findings and themes. Afterwards, we created a table (which is presented in the end of our manuscript), where we extracted from every reference the technique used, as well as our main observations.

Following the Introduction, this paper is organized as follows. In Section 2, we present fundamentals of risk-based continuous user authentication for mobile devices. In Section 3, we give an extensive overview of existing quantitative risk estimation approaches that could be possibly adopted in risk-based continuous user authentication solutions. Finally, the paper is concluded in Section 4.

## 2. Fundamentals of Risk-Based Continuous User Authentication Relying on Behavioral Biometrics for Mobile Devices

**Figure 1:**
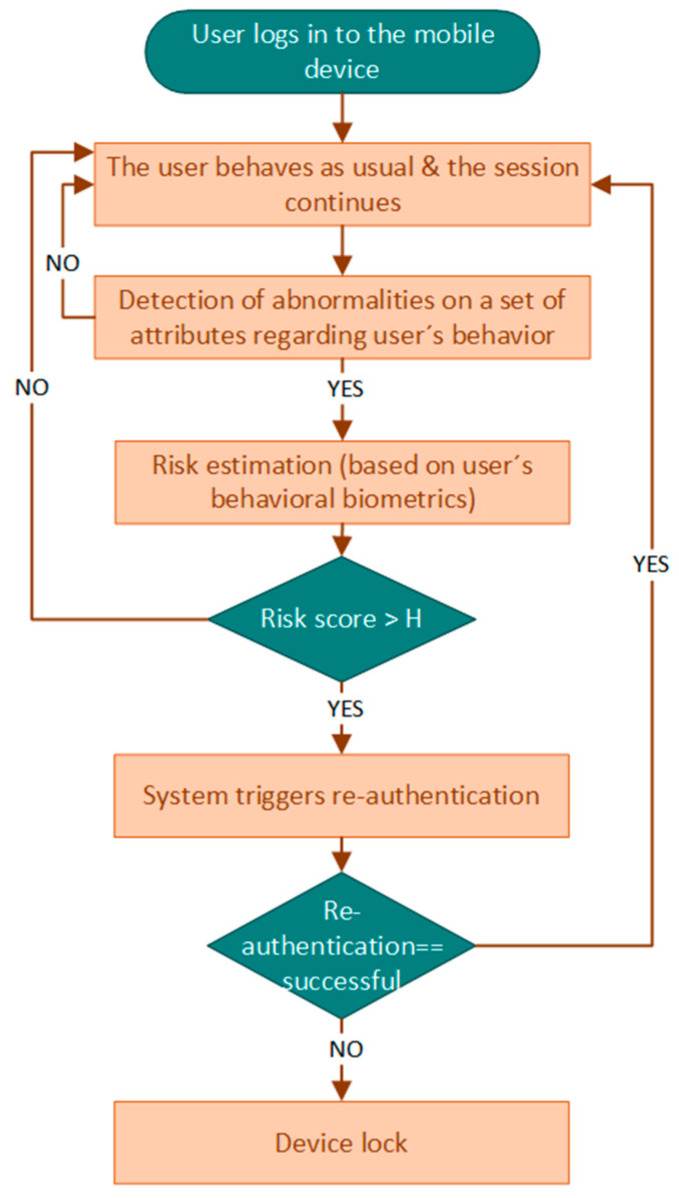
An overview of risk-based continuous user authentication relying on behavioral biometrics.

Risk-based continuous user authentication, a method relying on behavioral biometrics, has been shown to have the potential to further improve mobile authentication security without sacrificing usability (i.e., security and usability are often thought of as being contradictory) [12,21,22,23]. More specifically, risk-based continuous user authentication mechanisms manage to continuously verify the user’s identity during their entire interaction with the smartphone device, a task they perform based on a real-time risk score, as depicted in Figure 1. In particular, when an abnormality in a set of attributes regarding user’s behavior has been detected (i.e., event-driven approach), a real-time risk score is estimated. If this risk score is above an acceptable threshold (i.e., high risk score), re-authentication will be triggered. Re-authentication might rely on behavioral biometrics, verifying the authenticity of the user unobtrusively without the user needing to respond to an explicit authentication request.

Summarizing, the benefits of risk-based continuous user authentication whn relying on behavioral biometrics, they are as follows:

(i) to overcome the limitations of the conventional one-time authentication, in which user authentication only occurs at the start of the session, and afterwards, any future changes and/or abnormalities in user identity/behavior remain undetected;
(ii) to increase the efficiency of user authentication by triggering the verification process for user re-authentication only when it is actually required (i.e., real-time risk score above predefined threshold), minimizing the consumed resources;
(iii) to adapt the user re-authentication levels (i.e., uni-modal or multimodal authentication) autonomously in an on-the-fly approach based on the apparent risks (i.e., real-time risk score);
(iv) to re-authenticate users unobtrusively, based on their interactions with the device (i.e., beharioural biometrics), and thus address the security versus usability challenge in mobile user authentication.

However, it is worthwhile mentioning that, while behavioral biometrics have several advantages when used for user authentication, there are also limitations and potential risks that need to be taken into account. According to [11,24], behavioral biometrics are subject to false positives/false negatives. This is because behavioral biometrics rely on patterns of behavior to authenticate legitimate users, such as keystroke dynamics or mouse movement patterns. However, these patterns can change over time or might be affected by external factors such as environmental conditions, leading to false positives where legitimate users are denied access [11,24]. Similarly, behavioral biometrics can also result in false negatives, whereby unauthorized users are mistakenly granted access. This can happen if an attacker is able to mimic the user’s behavior [11,24]. On top of that, behavioral biometrics can also suffer from inconsistencies, such as when users are in a hurry, stressed, or distracted, which can lead to variations in their behavior that are difficult to capture and authenticate, leading again to false positives/false negatives. To overcome this limitation, the authors in [11,24] state that it is crucial to maximize the accuracy of the deployed algorithms. They additionally argue that since the user’s behavior and habits might change over time, authentication systems should also be able to adapt to these changes [11,24].

Another concern is user’s privacy and how this concern might affect user’s acceptance. Some users may be hesitant to adopt behavioral biometrics for authentication, as they may feel uncomfortable with the collection and use of their behavioral data for authentication purposes [11,24]. There are also potential security risks associated with the collection and storage of behavioral biometric data, as these data may be susceptible to hacking or misuse. Therefore, it is important to weigh the benefits and risks of behavioral biometric authentication and ensure proper security measures are in place to protect them. However, behavioral biometrics (e.g., gait, keystroke dynamics) remain less sensitive than physiological biometrics (e.g., fingerprints, iris) [11,24].

## 3. Quantitative Risk Estimation Approaches (QREAs)

**Figure 2:**
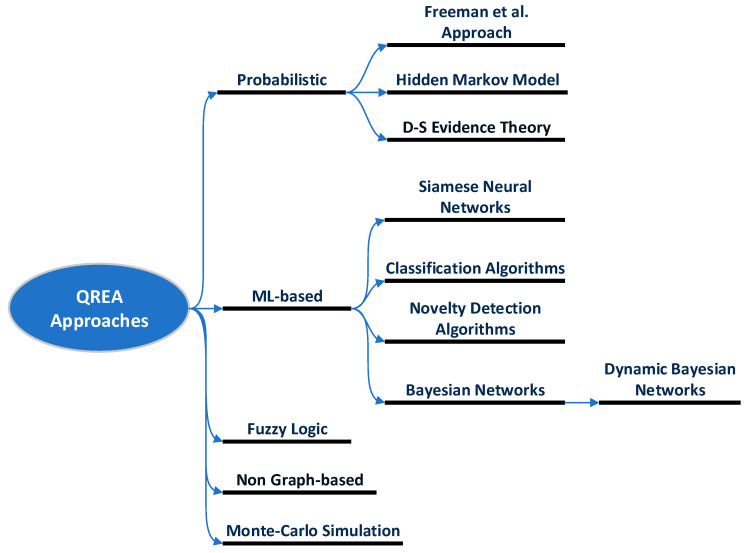
A taxonomy of Quantitative Risk Estimation Approaches. Freeman et al. [33].

This section gives an extensive overview of existing quantitative risk estimation approaches (QREA) found in the literature for: (i) risk-based user authentication on mobile devices, and (ii) other security applications, such as user authentication in web/cloud services, intrusion detection systems, etc., that could be possibly adopted in risk-based continuous user authentication solutions for smartphones. The target of this study is to provide a foundation for organizing research efforts toward the design and development of proper quantitative risk estimation approaches for risk-based continuous user authentication solutions for smartphones. The reviewed quantitative risk estimation approaches have been divided into the following five main categories, as also shown in Figure 2: (i) probabilistic approaches, (ii) machine learning-based approaches, (iii) fuzzy logic models, (iv) non-graph-based model, and (v) Monte Carlo simulation models.

### 3.1. Probabilistic QREA Approaches

#### 3.1.1. Freeman et al. Approach

In 2016, Freeman et al. [33] presented the first public probabilistic approach to derive a risk score for a login attempt in a web service in order to classify the login attempt into normal or suspicious, strengthening password-based authentication in this way. The underlying idea behind their proposed model is to exploit complementary data when a user tries to login to a web service, beyond the validation of just their credentials. These complementary data can be obtained from the HTTP session logs of authentication sessions established between the web server and the user such as the IP address, timestamp, and cookies. After this, they are compared against stored users’ login history data through a carefully designed statistical approach.

The model proposed by Freeman et al. [33] calculates the risk score for a login attempt in a web service *S* for a user *u* and a given feature set (*FV* 1, …, *FVd*), where *d* is the total number of features characterizing a login attempt (e.g., IP address, timestamp, browser, etc.) as follows:

(1) $$S_{u}\left(FV\right)=\left(\prod_{k=1}^{d}\frac{p\left(FV^{k}\right)}{p\left(FV^{k}\left|u,legit\right.\right)}\right)\frac{p\left(u\left|attack\right.\right)}{p\left(u\left|legit\right.\right)},$$

where $p\left(FV^{k}\right)$ is the probability of a feature value to appear in the global login history, and $p\left(FV^{k}\left|u,legit\right.\right)$ is the probability of a feature value to appear in the legitimate user’s login history.

The user login probability $p\left(u|legit\;\right)$ derives from the proportion of the user logging in as follows:

(2) $$p\left(u|legit\;\right)=\frac{number\;of\;user\;logins}{number\;of\;all\;logins},$$

On top of that, attack data might be also taken into consideration by the statistical model of Freeman et al. [33] for more accurate login attempt classification (normal/suspicious). However, Wiefling et al. [27] highlighted that use cases without attack data are more common in real-world applications, especially for medium- and small-sized websites that have limited storage and computing capacity. In this case, and considering that all users are equally likely to be attacked, Wiefling et al. considered the attack probability as follows: $p\left(u\left|attack\right.\right)=\frac{1}{\left|U\right|}$, where *U* is the set of users and *u* ∈ *U* [27].

Wiefling et al. [27] extended Equation (1) in order to include the attack probability for the selected feature values:

(3) $$S_{u}\left(FV\right)=\left(\prod_{k=1}^{d}p\left(attack\left|FV^{k}\right.\right)\frac{p\left(FV^{k}\right)}{p\left(FV^{k}\left|u,legit\right.\right)}\right)\frac{p\left(u\left|attack\right.\right)}{p\left(u\left|legit\right.\right)}$$

where $p\left(attack\left|FV^{k}\right.\right)$ comprises the total number of feature value occurrences in the failed login attempts.

**Figure 3:**
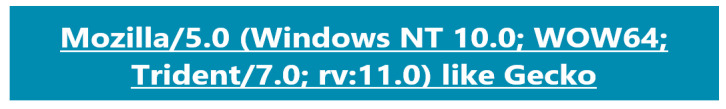
An example of *useragent* string when using Internet Explorer 11.0 (i.e., Mozilla: MozillaProductSlice, this is only used for historical reasons; 5.0: Mozilla version; Windows NT 10.0: Operating System; WOW64 (Windows-On-Windows 64-bit). A 32-bit application is running on a 64-bit processor; Trident: Layout engine for the Microsoft Windows version of Internet Explorer; 7.0: Trident version; rv:11.0: Browser version).

For their prototype implementation, Freeman et al. [33] chose the features values of: (i) IP address and (ii) *useragent* string (an example of which is given in Figure 3). The authors selected these particular features because they: (a) are attached to real users (and not bots) as their primary goal is to improve detection of non-bot account compromise; (b) admit natural hierarchies, and thus the authors will be able to apply certain techniques to calculate the probabilities required by their statistical model in the presence of sparse data; and (c) can be obtained easily from the LinkedIn dataset which the authors used to build their model. Freeman et al. [33] highlight that, although in practice IP and *useragent* are correlated, and thus the independence assumption that allows us to derive Equation (1) does not hold in practice, this correlation is not strong enough to prevent the two features from providing complementary signals to their model.

#### 3.1.2. Hidden Markov Model

Hidden Markov Models (HMMs) can be used for risk estimation in various applications, such as finance, healthcare, and security [34,35]. HMMs are a type of probabilistic model that can be used to model sequential data, where the underlying states are hidden and only observable through a set of outputs [36]. In the context of risk estimation, HMMs can be used to model the underlying states of a system and the observations that result from these states [34,35]. For example, in a security application, the states might represent different levels of risk (e.g., low, medium, and high), while the observations might be the sensor readings or other inputs that are used to estimate the risk level [34,35].

Once an HMM is trained on historical data, it can be used to estimate the probability of different risk levels in a way that accounts for the current set of observations [34,35]. This can be useful for real-time risk assessment and decision making in various applications. One advantage of using HMMs for risk estimation is that they can handle sequential data and incorporate temporal dependencies between observations [36]. Additionally, HMMs can handle missing or incomplete data, making them robust to noisy or imperfect sensor readings [36]. Overall, HMMs can be a powerful tool for risk estimation in various applications as they provide a flexible and probabilistic framework for modeling complex systems and making real-time predictions based on incomplete and uncertain information [34,35].

##### Arnes et al.

Arnes et al. [35] proposed the use of a real-time risk assessment approach for intrusion detection systems based on information derived from network sensors. In their proposed risk assessment method, the risk was dynamically evaluated using Hidden Markov Models (HMMs). In this way, their proposed mechanism managed to handle the data from sensors with a different degree of trustworthiness. The authors defined the degree of trustworthiness of a sensor in terms of its false positives and false negatives. The main advantage of their study is that enables data aggregation from various sensors with different weighting according to their trustworthiness. By using HMMs, Arnes et al. [35] are able to identify the most possible state probability distribution of monitored objects, considering the degree of trustworthiness of the IDS. Regarding the types of sensors employed in their monitoring architecture, the authors assume that they can provide standardized output as required by the model parameters.

Arnes et al. [35] considered two main entities in their system: (i) the sensor, which can be any information-gathering device and/or program, such as virus detectors, honeypots, logging systems, network sniffers, etc., which are used to collect information regarding the security state of monitored objects; and (ii) the agent, which is a computer program that can perform a certain degree of autonomous actions and whose its main task is the sensor data collection and aggregation. These sensor data derive from a set of sensors that monitor a set of objects to perform real-time risk assessment. As such, Arnes et al. [35] propose a multi-agent system architecture consisting of multiple agents that monitor objects in a network using sensors. In such networks, agents have the capabilities to communicate and cooperate with other agents. Arnes et al. [35] selected the multi-agent architecture for its scalability and flexibility, as well as for supporting distributed automated responses.

In this study, $O=\left\{o_{1},\;o_{2},\;.\;.\;.\right\}$ is the set of objects that an agent monitors. This set of objects represents the part of the network that the agent is responsible for, and the security state of each object is described using discrete-time Markov chains. Assuming that each object consists of N states, denoted as $S=\left\{s_{1},\;s_{2},\;.\;.\;.\;,\;s_{N}\right\}$, the security state of an object is not constant. On the contrary, it changes over time, moving between the states in S. Thus, the sequence of an object’s states is denoted as $X=x_{1},\;x_{2},\;.\;.\;.\;,\;x_{T}$, where $x_{t}\in S$ is the object’s security state at time t. For the purpose of their study [35], Arnes et al. considered that the object’s state space can be represented by a general model consisting of three states: (i) Good (G), (ii) Attacked (A) and (iii) Compromised (C), e.g., S = {G, A, C}. State G indicates that the object is running securely and that it is not subject to any kind of attack. The authors assume that objects are constantly vulnerable to attacks, even in state G, and that when an attack is initiated, the security state of this object will move from G to A. Thus, state A indicates that the object is subject to an ongoing attack, which probably affects its general behavior regarding security. Finally, state C indicates that the object has been successfully compromised by an attacker, being subject to any kind of confidentiality, integrity and/or availability breaches. Afterwards, Arnes et al. [35] modeled the objects using HMMs, defined by $\lambda =\left\{P,Q,\;\pi \right\}$, where

$P=\left\{p_{ij}\right\}$ is the state transition probability distribution matrix for object o, where $p_{ij}$ represents the probability that object o will transfer from state $s_{i}$ into state $s_{j}$ next, denoted as $p_{ij}=P\left(x_{t+1}=s_{j}|x_{t}=s_{i}\right),1\leq i,j\leq N.\;$To be able to estimate P for real-life objects, the authors state that they might use either statistical attack data from production or experimental systems or the subjective opinion of experts. However, the deployment of learning algorithms may give a better estimate of P over time [35].

Additionally, $Q=\left\{q_{j}\left(l\right)\right\}$ is the observation symbol probability distribution matrix for object o in *s_j_*, whose elements are $q_{j}\left(l\right)=P\left(y_{t}=v_{l}|x_{t}=s_{i}\right),1\leq j\leq N,\;1\leq l\leq M$. Arnes et al. [35] considered $q_{j}\left(l\right)$ in Q as the probability that a sensor will send the observation $v_{l}$ at time t, given object *o* in $s_{j}$ at t. Consequently, Q actually indicates the sensor’s trustworthiness, i.e., sensor’s false-positive and false-negative effects on the agents’ risk assessments.

Finally, $\pi =\left\{\pi_{i}\right\}$ is the object’s o initial state distribution. Hence, $\pi_{i}=P\left(x_{1}=s_{i}\right)$ is the probability that $s_{i}$ was the initial state of o.

Afterwards, Arnes et al. [35] constructed their quantitative risk assessment approach following the terminology used in [37]. According to [37], risk was measured in terms of consequences and likelihood. In particular, a consequence was that the outcome of an event and could be qualitative or quantitative, while the likelihood was a description of the probability of that event to happen. To perform dynamic risk assessment, the authors needed to map C : S → ℝ, describing the expected cost for each object due to loss of integrity, confidentiality, and availability. Then, the total risk $R_{t}$ for an object at time t is defined as:

(4) $$R_{t}=\overset{N}{\underset{i=1}{\sum }}R_{t}\left(i\right)=\overset{N}{\underset{i=1}{\sum }}\gamma_{t}\left(i\right)C\left(i\right)$$

where $\gamma_{t}\left(i\right)$ is the probability that object o is in security state $s_{i}$ at time t, and $C\left(i\right)$ is the associated cost value.

In order to perform real-time risk assessment for an object o, the agent that is responsible for this object has to dynamically update the object’s state probability $\gamma_{t}=\left\{\gamma_{t}\left(i\right)\right\}$. Given an observation $y_{t}$, and the HMM λ, Arnes et al. [35] proposed a particular algorithm (i.e., Algorithm 1 in [35]) based on which the agent can update the state probability $\gamma_{t}$ of an object. The complexity of the algorithm is O(N^2^).

To illustrate the theory, Arnes et al. [35] deployed a typical home office network (HON) and performed a real-time risk assessment on it. The typical home office network consisted of a laptop using WLAN, a cell phone connected to the laptop using Bluetooth, a stationary computer with disk and printer sharing, and an Internet router/WLAN access point (AP). The AP was considered to be equipped with a network monitoring sensor that monitored traffic between the internal hosts (a network IDS) and the outside network, while each of the objects (hosts) in the HON was equipped with a sensor that produced and processed log files and checked system integrity (i.e., a host IDS).

Overall, Arnes et al. [35] introduced an HMM-based approach for assessing risks in real-time. The approach involves consolidating data from various sensors and assigning different weights to each sensor based on reliability. The proposed model operates in discrete time and depends on the periodic transmission of sensor data, which necessitates the sampling of alert information. To make the method more practical for use in real-world scenarios, Arnes et al. [35] suggested further development using continuous-time models in order to be able to handle highly variable alert rates from multiple sensors. Furthermore, Arnes et al. [35] proposed extending this approach to a multi-agent system with automated response capabilities, enabling agents to evaluate and respond to the risk level for several objects. Their proposed risk assessment showed promising results (i.e., false-positive and false-negative rates), and it could be the basis for automated response IDSs. However, more mechanisms need to be deployed and combined to provide a system that will be able to relate detected security incidence to an appropriate response based on the underlying risk assessment model [35]. The authors give some indications on how their study can be extended into a multi-agent system with automated responses, where agents are responsible for assessing and responding to the risk for several objects.

##### Chen et al.

**Figure 4:**
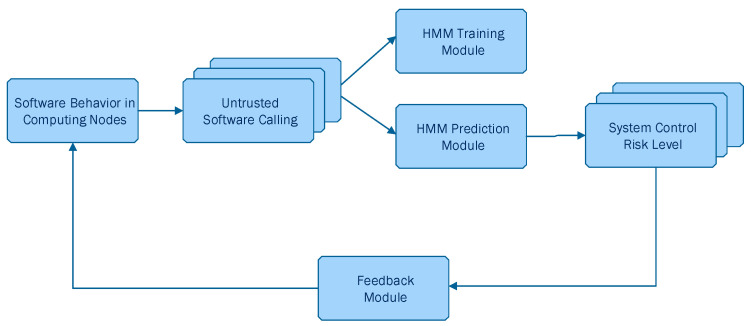
A risk assessment approach based on software behavior, Chen et al. [34].

On the other hand, Chen et al. [34] discussed the weaknesses of traditional risk assessment methods in terms of the subjectivity of experts/assessors and inaccuracy of vulnerability detection, leading to the obtention of non-quantitative and unreliable results, proposing instead an approach for quantitative risk assessment based on software behavior using HMMs. Their proposed system architecture is depicted in Figure 4. Chen et al. [34] followed a similar approach as Arnes et al. [35], with the difference being that Chen et al. [34] considered four security states, namely Low (L), General (G), Medium (M) and High (H), denoted as $S=\left\{s_{1},\;s_{2},\;.\;.\;.\;,\;s_{N}\right\},\;where\;n=4$. In addition, Chen et al. [34] used Baum–Welch algorithm, which is the most widely used unsupervised learning algorithm in HMM for dynamically updating the object’s state probability $\gamma_{t}=\left\{\gamma_{t}\left(i\right)\right\}$.

Chen et al. [34] generated observable sequences from data derived from daily statistics to train their HMM. Afterwards, they considered four hosts to assess the risk control level of the system. Each host had set up the whitelist mechanism to identify if the called software was or not trustworthy. In the case that is untrusted, the host continued to detect whether the untrusted software had been successfully invoked. This test simulated the user’s software calling behavior and randomly invoked trusted/untrusted software. The authors suggested that their approach showed credible risk assessment results in a quantitative way and can be applied in the actual risk assessment process in other applications.

#### 3.1.3. D–S Evidence Theory

D–S Evidence Theory, also referred to as D–S Theory, which was proposed by Dempster and extended by Shafer, is a probabilistic approach to reasoning under uncertainty that can be used for various tasks, including risk assessment for various applications [38]. The D–S evidence theory is relevant and useful for risk assessment and other applications due to its following characteristics [38]:

- Ability to handle uncertainty: The D–S theory can handle uncertain and incomplete information, making it useful in situations where traditional probability theory may not be suitable.
- Incorporation of multiple sources of evidence: The D–S theory allows for the integration of evidence from multiple sources, even when they may be conflicting or inconsistent, making it well-suited for situations where there are multiple sources of information.
- Flexibility in representation: The D–S theory provides a flexible framework for representing uncertainty and making decisions based on evidence, making it adaptable to a wide range of applications.
- Transparent reasoning process: The D–S theory provides a transparent reasoning process that allows users to trace the origins of their beliefs and decisions.
- Robustness to outliers: The D–S theory is robust to outliers or noise in the data, making it well-suited for applications where there may be inaccuracies or errors in the data.

Overall, the D–S evidence theory can be a valuable tool for risk assessment and other applications in which uncertainty and multiple sources of evidence need to be taken into account [38]. It provides a flexible and transparent framework for reasoning under uncertainty, making it useful for a wide range of applications [38].

D–S evidence theory is based on the concept of belief functions, which are used to represent the degree of belief or disbelief in a particular hypothesis or statement. Belief functions can be combined using Dempster’s rule of combination, which considers the degree of conflict or overlap between different belief functions. The resulting belief function can then be used to calculate the final risk score for a given input. D–S theory is a commonly used tool in solving complex problems with uncertainties caused by ignorance [38]. In the following, we introduce the part of D–S theory related to the online risk assessment model, as has been proposed by Mu et al. [38].

The Frame of Discernment Θ is a finite hypothesis space consisting of mutually exclusive propositions for which the information sources can provide evidence, while $2^{\Theta }$ denotes its powerset [38]. Then, the mass function m, or also known as the Basic Probability Assignment (BPA) is defined as follows:

(5) $$m:\;2^{\Theta }\to \left[0,\;1\right]\;m\left(\phi \right)=0\;\underset{V\subseteq \Theta }{\sum }m\left(V\right)=1$$

where ϕ is an empty set, $m\left(V\right)$ is the proportion of all available and applicable evidence that supports the claim that a specific element of the universal set X belongs to the subset V. Subset V is called focal element of m when $m\left(V\right)>0$.

Afterwards, Dempster’s Rule of Combination calculates the joint support contribution, reducing uncertainties this way by combining different pieces of evidence together. The rule is given by the combined mass function $m=m_{1}⊕m_{2}⊕\ldots ⊕m_{n}$, as follows:

(6) $$m\left(\phi \right)=0\;m\left(V\right)=\frac{\sum_{\cap V.=V}\prod_{1\leq q\leq n}m_{q}\left(V_{j}\right)}{\sum_{\cap V.\neq \phi }\prod_{1\leq q\leq n}m_{q}\left(V_{j}\right)}$$

where the combination operator ⊕ is called orthogonal summation [38].

##### Mu et al.

**Figure 5:**
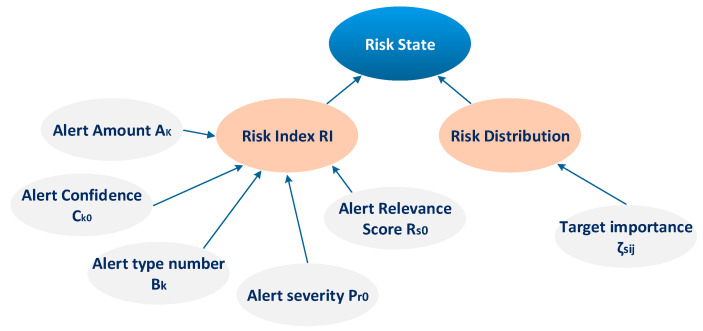
Online risk assessment model proposed by Mu et al. [38].

For their proposed online risk assessment model, Mu et al. [38] considered two important notions: (i) the Risk Index (RI), and (ii) the Risk Distribution, as depicted in Figure 5. According to Mu et al. [38], RI is the dangerous degree to a protected target. This risk is caused by an intrusion scenario and it might be considered in three cases: (1) the probability that an attack successfully compromises an asset of the system; (2) the probability that an irregular behavior, detected by IDS, is an actual attack given the fact that only a true and effective attack is capable of causing a true threat to a protected target; and (3) the probability that the severity caused by an attack, as attacks with different degrees of severity might result in different threats and damages to a protected target. Figure 5 presents in detail the factors that influence the RI in [38]. On the other hand, the Risk Distribution represents the spectrum (i.e., thresholds) of the low, medium and high risk that a target can tolerate. The Risk Distribution of a target is determined by the importance of the target for the whole system. This value is usually evaluated by a subjective approach of an administrator [38].

Their proposed online risk assessment model is presented in Figure 5. Their model fuses five assessment factors (i.e., Alert Amount A_k,_ Alert Confidence C_k0_, Alert type number B_k_, Alert severity P_r0_, and Alert Relevance Score R_s0_) to compute RI using D–S evidence theory. In their proposed model, these factors are acquired from the alert confidence learning, the alert verification and alert correlation. Meanwhile, the importance of the target is taken into consideration for the determination of the target risk distribution. Finally, the final risk score, which in the proposed model is the risk state of the target, can be defined by the position of RI in the final overall risk distribution of the target.

Mu et al. [38] performed some experiments, deploying IDAM&IRS and Snort 2.0 IDS on the subnet (xxx.71.75.130-xxx.71.75.180) in their laboratory that was connected to the Internet to test and evaluating their proposed online risk assessment model. The authors also installed Norton Internet Security 7.0 and BlackICE PC Protection on some hosts in xxx.71.75.130-xxx.71.75.180. In their experiment, there were four types of network servers, namely Http Proxy, Ftp, Web and Database in the subnet, while the main operating systems included Windows 2000, Windows XP, Windows 2003 server, and Linux. According to their results, the deployment of their proposed risk assessment model enabled IDAM&IRS to tolerate IDS false positive alerts, establishing in this way the basis for effective intrusion response decision making [38].

### 3.2. ML-Based RBA Models

#### 3.2.1. Siamese Neural Networks

**Figure 6:**
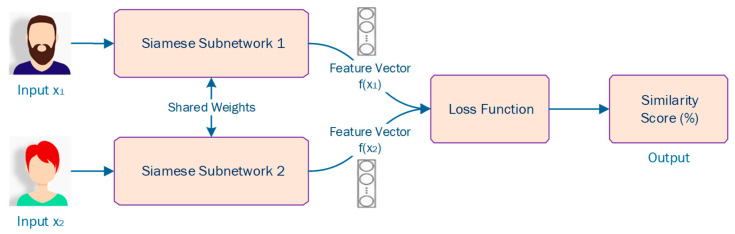
An example of a Siamese Neural Network (SNN).

A Siamese Neural Network (SNN) is a group of neural networks that comprise two or more identical subnetworks, as depicted in Figure 6. The term ‘identical’ means that these subnetworks have the exact same configuration (i.e., similar parameters and weights). On top of that, parameter updating is reflected across both subnetworks. Then, the SNN extracts the feature vectors of the two or more subnetworks and, using a loss function, outputs the similarity score of the two or more inputs by comparing their feature vectors.

Since, in most cases, the training of SNNs requires pairwise learning, cross entropy loss cannot be used. Typically, triplet loss or contrastive loss are popular loss functions used in training SNNs [38,39,40,41]. Triplet loss comprises a loss function in which there is an anchor or baseline input that can be compared to a positive input (i.e., truth) and a negative input (i.e., false) [42]. The main idea is that this loss function tries to minimize the distance from the anchor or baseline input to the positive input and maximize the distance from the anchor or baseline input to the negative input. To do this, it follows Equation (7):

(7) $$L\left(A,P,N\right)=\max\left(\|f\left(A\right)-f{\left(P\right)\|}^{2}-\|f\left(A\right)-f{\left(N\right)\|}^{2}+a,0\right)$$

where a is a *margin* term used to “stretch” the distance differences between similar and dissimilar pairs in the triplet, and $f\left(A\right)$, $f\left(P\right)$, and $f\left(N\right)$ are the feature embeddings for the anchor, positive and negative inputs [42].

**Figure 7:**
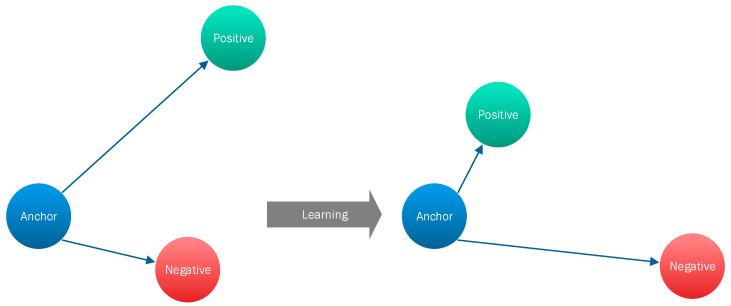
An example of the training process of a Siamese Neural Network (SNN) using triplet loss.

During the training process, an input triplet (i.e., anchor input, negative input, positive input) is fed into the SNN model as a single sample. The aim of this is that the distance between the anchor and positive inputs should be smaller than the distance between the anchor and negative input [42], as depicted in Figure 7.

On the other hand, the popular and commonly used nowadays loss function contrastive loss comprises a distance-based loss contrasted with the conventional error-prediction losses [42]. This loss is used to learn embeddings in which two identical points will present a low Euclidean distance, while two unidentical points will present a large Euclidean distance [42]. To do this, it follows the following equation:

(8) $$\left(1-Y\right)\frac{1}{2}{\left(D_{W}\right)}^{2}+Y\;\frac{1}{2}{\left\{\max\left(0,margin-D_{W}\right)\right\}}^{2}$$

where $D_{W}$ is the Euclidean distance:

(9) $$\sqrt{{\left\{G_{W}\left(X_{1}\right)-G_{W}\left(X_{1}\right)\right\}}^{2}}$$

where $G_{W}$ is the similarity score (i.e., output of SNN) for one image.

Compared to traditional neural networks as well as to other machine learning classifiers, SNNs’ main advantage is that they are one-shot learning classifiers which means that they can classify new classes of data without training the network again. This is because they learn a similarity function, rather than classify entries based on particular features. On top of that, they work well as an ensemble with the best or most efficient classifier. Additionally, its learning mechanism is fairly different from conventional classification. Indeed, simple averaging of the SNN output with the output of a conventional classifier can perform much better compared to the average of two correlated supervised models [42]. SNNs are widely used in image and text similarity, as well as signature verification [38,39,40]. Regarding risk estimation, SNNs can potentially be used to estimate risk by comparing features of different data points and identifying patterns that may indicate a higher or lower risk [38,39,40]. For example, in financial risk assessment, Siamese networks could be used to compare the features of different financial transactions and identify patterns that may indicate fraud or high-risk transactions [38,39,40]. However, it’s important to note that the effectiveness of SNNs for risk estimation would depend on the quality and relevance of the input data, as well as the specific problem being addressed [38,39,40].

##### Acien et al.

The SNNs have been widely used for image classification or handwritten signature verification in the literature. However, most recently SNNs have begun to be used for behavioral biometric-based user authentication [38,39,40]. In particular, Acien et al. [40] developed their TypeNet model based on a Siamese Recurrent Neural Network (RNN) and evaluated the effectiveness of keystroke dynamics as a behavioral biometric for authenticating 100 K users typing free-text, where the amount of data per user is limited, a common scenario in free-text keystroke authentication. The authors utilized the Aalto University keystroke database [40] in their experiments and obtained promising results with an equal error rate of 4.8%, a result obtained using only 5 enrollment sequences and 1 test sequence per user with 50 keystrokes per sequence for 1 K test users (a sample size comparable to previous studies). When the number of test users was increased to 100 K with the same amount of data per user, the performance degraded relatively by less than 5% equal error rate compared to 1 K test users, demonstrating the potential for scalability to large numbers of test users, which are representative of real-world security scenarios. According to the authors, this is the largest free-text keystroke database collected, featuring more than 136 M keystrokes from 168 K users.

To train the Siamese Recurrent Neural Network (RNN), Acien et al. [40] fed the network with two inputs consisting of two free-text keystroke sequences from either the same user or different users. During the training phase, the RNN learned to distinguish between the pairs of free-text keystroke sequences and then transformed this information into an embedding space. In this space, the embedding vectors (outputs of the RNN model) will be close in proximity when both free-text keystroke sequences belong to the same user (referred to as genuine pairs) and far apart when they do not belong to the same user (referred to as impostor pairs). For this, Acien et al. [40] used a contrastive loss function specifically defined for this task. This had been proposed by Taigman et al. [43] in 2014. If $\left(x_{i},x_{j}\right)$ is a free-text keystroke sequences pair that is provided as input to the RNN model, then, according to [43], the contrastive loss determines the Euclidean distance between the model outputs (i.e., embedding vectors) as follows:

(10) $$d_{E}\left(x_{i},x_{j}\right)=\|f\left(x_{i}\right)-f\left(x_{j}\right)\|,$$

where $f\left(x_{i}\right)$ and $f\left(x_{j}\right)$ are the model outputs for the inputs $\left(x_{i},x_{j}\right)$, respectively. The model will learn to make this distance $d_{E}$ small (~0) in case of a genuine input pairs and large (~α) in case of impostor input pairs by computing the contrastive loss function L as follows:

(11) $$L=\left(1-L_{ij}\right)\frac{d_{E}^{2}\left(x_{i},x_{j}\right)}{2}+L_{ij}\frac{max^{2}\left\{0,\alpha -d_{E}\left(x_{i},x_{j}\right)\right\}}{2}$$

where $L_{ij}$ is the label associated with every input pair:

$$L_{ij}=\left\{\begin{array}{c} 0,\;for\;genuine\;pairs\;\\ 1,\;for\;impostor\;pairs \end{array}\right.$$

Additionally, α≥0 a distance that represents the maximum margin between genuine and impostor distances.

After training the RNN model, Acien et al. [40] tested their model, authenticating users by comparing samples that belong to one of the users in the test set $x_{g}$, with a sample $x_{q}$ from either the same user (i.e., genuine match) or another user in the test set (i.e., impostor match). Then, they computed the final test score (or similarity score) by averaging the Euclidean distances $d_{E}$ between each embedding vector $f\left(x_{g}\right)$ and the embedding vector $f\left(x_{q}\right)$ as follows:

(12) $$score=\frac{1}{G}\overset{G}{\underset{g=1}{\sum }}d_{E}\left(f\left(x_{g}\right),f\left(x_{q}\right)\right)$$

where *G* is the number of enrollment samples per user. Considering that each user has a total of 15 sequences (i.e., enrollment samples per user), the authors keep 5 sequences (i.e., 5 genuine test scores) per user as test set and let *G* vary between 1>G> 10 to evaluate the performance as a function of number of enrollment sequences.

It is important to highlight that Acien et al. [40] compared the performance of their proposed model with state-of-the-art studies found in the literature and showed that TypeNet performs more efficiently and effectively in terms of Equal Error Rate (EER) and computational time. This is an important advance for behavioral biometric-based user authentication and also demonstrates the potential use of SNN in risk-based user authentication based on behavioral biometrics. In particular, Equation (12), used by Acien et al. to calculate the final test score (or similarity score), could be used to estimate the real-time risk score when deploying risk-based user authentication on smartphones as the final test score, a process which takes into consideration behavioral biometrics, such as free-text keystroke sequences. This is similar to the risk estimation process in risk-based continuous user authentication, which relies on behavioral biometrics for calculating the risk score, as mentioned in Section 2.

#### 3.2.2. Classification Algorithms

Classification algorithms comprise a large category of machine learning algorithms, whose aim is to identify which of a set of categories a new observation belongs to. For example, typical classification problem is to assign a certain email to the “spam” or “non-spam” class, or, in our case, to assign an activity and/or event to the “low risk”, “medium risk” or “high risk” class. Classification algorithms have been widely used in the literature for identifying the risk associated with a particular event or action deploying risk-based user authentication [22,27,44,45,46,47,48,49].

##### Misbahuddin et al.

**Figure 8:**
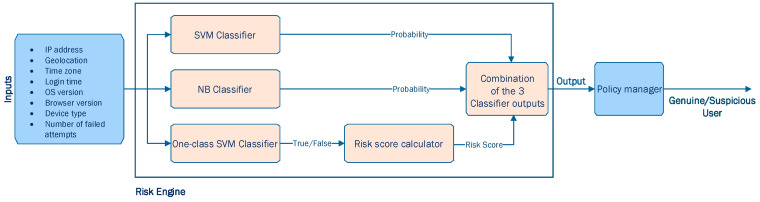
The architecture of the Risk Engine proposed in [49].

In [49], Misbahuddin et al. proposed the design of a risk engine capable of analyzing specific attributes of user’s past login records (i.e., IP address, geolocation, time zone, login time, OS version, browser version, device type, and number of failed attempts) and generating a proper pattern using classification algorithms to determine the risk score every time the user sign-in. Their proposed risk engine combined three different machine learning classification algorithms (i.e., Support Vector Machine (SVM), one-class SVM, and Naïve-Bayes (NB) classification algorithms), as depicted in Figure 8.

In their application [49], Misbahuddin et al. aim to classify whether a user that tries to log in into the system is genuine or suspicious and trigger relevant actions, respectively. According to their design, the SVM and NB classifiers are expected to output a probability, and more specifically to output the probability of the user being fraudulent. On the other hand, a one-class SVM classifier is expected to output a Boolean value (i.e., “True” indicating that the user is genuine or “False” indicating that the user is suspicious). In case that the output is “False”, Misbahuddin et al. propose the following Equation (13) to estimate the probability of this user being suspicious, also known as a risk score:

(13) $$Risk\;score=\overset{n}{\underset{i=1}{\sum }}user_{parameter_{value_{i}}}\times user_{parameter_{weight_{i}}}$$

where,

$$user_{parameter_{value}}=\left\{\begin{array}{c} 0,\;if\;user\;behavior\;exists\;in\;\mathrm{user}’s\;\mathrm{past}\;\mathrm{login}\;\mathrm{records}\;\\ 1,\;if\;user\;behavior\;does\;not\;exist\;in\;\mathrm{user}’s\;\mathrm{past}\;\mathrm{login}\;\mathrm{records} \end{array}\right.$$

After evaluating the impact that each user_parameter would have in determining potential risk for their system, Misbahuddin et al. proposed the user_parameter_weight, as appears in Table 1 (from least severe potential risk to most severe potential risk). Afterwards, the Risk Engine combined the 3 different outputs from the 3 classifiers and assigned different risk levels according to Table 2. Table 2 also presents the required further actions that the user should perform based on the identified risk level.

Misbahuddin et al. [49] implemented their proposed mechanism using Android, java and R programming. On top of that, they tested their scheme, modifying every time certain features during user’s login. The authors provided valuable information regarding the performance of their 3 classifiers [49]. In particular, they highlighted that, in order for SVM and NB classifiers to be efficient, they need to be trained with sufficient data from both classes (i.e., genuine user class and suspicious user class). In cases that this condition cannot be assured and data of both classes may not be available, which is very frequent in real-world applications, Misbahuddin et al. [49] proposed that researchers to deploy a one-class SVM classifier. Finally, the proposed mechanism also ensured usability in addition to device security as a genuine user is not required to perform multiple factors of authentication methods to effectively prove their authenticity, while suspicious users must perform various authentication methods depending on the risk level associated with their action [49].

In [48], the authors tested and evaluated the performance of SVM, NB, Decision Tree (DT), and k-NN; a set of the most popular classification algorithms for risk-based authentication. These classification algorithms were trained and tested over the HuMIdb dataset [50,51], which, to the best of authors’ knowledge, is the most recent and publicly available dataset for behavioral user authentication. Afterwards, these classification algorithms were evaluated in terms of: accuracy, precision, recall, and F1-score. It was noticed that, during the training process, generated models were used to become very closely related to training data with certain training features and thus, perfect scores (i.e., 100%) were achieved by the models. As such, derived from overfitted models, these evaluation results cannot be considered reliable; on the contrary, they are strongly reliant and biased towards specific features of the training data. Therefore, the classification algorithms tested in this study [48] should not be considered as proper algorithms for deploying risk-based user authentication mechanisms. To overcome the challenge of overfitting, the authors considered the concept of novelty detection, training and testing the following novelty detection algorithms: OneClassSVM, Local Outlier Factor (LOF), and KNN_average (i.e., KNN configured properly for novelty detection). All of them demonstrated a level of high performance. To the best of authors’ knowledge, this was the first time that novelty detection algorithms have been considered for risk-based user authentication. The findings in [48,52] highlighted the advantages of one-class novelty detection algorithms, presented in detail in Section 3.2.3, over popular machine learning classifiers for risk-based user authentication.

#### 3.2.3. Novelty Detection Algorithms

**Figure 9:**
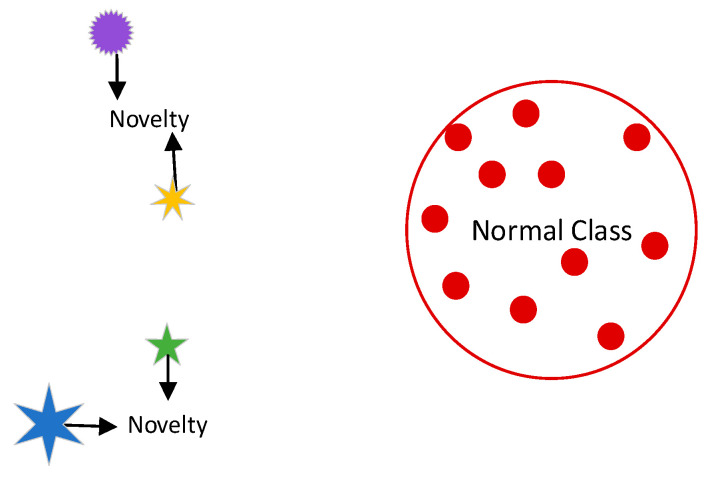
Visualization of novelty detection concept.

Smartphone device user authentication requires the ability to determine if a new observation, such as a log-in from an unfamiliar location, belongs to the same distribution as existing observations (i.e., is an inlier) or to a different distribution (i.e., is an outlier) [53]. The process of detecting such anomalies is referred to as novelty detection and is also known as semi-supervised anomaly detection. This is because, in novelty detection, the training data are not contaminated by outliers and the goal is to determine if a new observation is an outlier, as illustrated in Figure 9 [53].

In particular, risk-based user authentication, relying on behavioral biometrics, normally involves single-user smartphone devices where it is necessary to differentiate between a known legitimate user and an unknown malicious user [52]. In this context, novelty detection algorithms, also known as one-class classifiers [54,55,56], have gained interest among researchers due to their potential advantages in user authentication based on behavioral biometrics. Antal et al. [57] compared the performance of one-class classifiers and multi-class classifiers for keystroke-based user authentication on smartphone devices and found that multi-class classifiers outperformed one-class classifiers, with a difference of 4% error rate. On the other hand, Gupta et al. [54] conducted a comprehensive review of the state-of-the-art one-class classifiers and analyzed their collected dataset, before selecting four classifiers for their IDeAuth system: Local Outlier Factor (LOF), Minimum Covariance Determinant (MCD), Isolation Forest (IF), and one-class Support Vector Method (SVM). Their selection criteria included the classifier’s efficiency for platforms with limited computing power, diversity of the learning paradigm of the classifiers, nominal memory consumption, and ability to handle similar sensory data. Their proposed scheme, IDeAuth, achieved a Half Total Error Rate (HTER) of approximately 4% through decision-level fusion, with an improvement of approximately 1% versus the best-performing MCD classifier. The HTERs for the MCD, LOF, IF, and SVM classifiers, trained with 20 Singular Value Decomposition (SVD) components, were 5.25%, 6.89%, 7.28%, and 9.06%, respectively. Shen et al. [58] used one-class SVM, Neural Network, and KNN-based one-class classifiers for user authentication based on mouse usage patterns, reporting HTERs of approximately 8%, 15%, and 15%, respectively, on a dataset of 5550 mouse operation samples collected from 37 subjects. On top of that, they also argued that one-class classifiers are more suitable for user authentication in real-world applications [58].

Furthermore, according to [56], one-class classifiers, particularly the one-class SVM, have been utilized to address a range of authentication challenges, including touch and mouse dynamics recognition, smart-stroke, and face recognition. In [59], Antal et al. developed an authentication model based on swipe gestures using four one-class classifiers, including the Parzen density estimator, kNN_average, Gaussian mixtures method, and Support Vector Data Description method. The swipe gestures and micro-movements of the device were collected under a controlled environment while participants were completing a psychological questionnaire. The kNN_average and Parzen density estimator achieved the lowest mean Equal Error Rates (EER), i.e., 0.024 and 0.023, respectively, after combining the decisions from multiple swipe gestures.

In fact, the main benefit of one-class novelty detection algorithms over other types of ML algorithms, especially classification algorithms, is that for model training, they only require genuine samples, and not samples from the impostors’ class. The fast progress in the data acquisition quality of mobile computing devices, along with the general lack of available data for behavioral biometrics, constitute novelty detection, a semi-supervised method and suitable approach for risk-based user authentication relying on behavioral biometrics. Classification algorithms, which are usually supervised models, are challenging to use in real-world user authentication applications as there are not enough negatively labeled samples available per user. In [48], the novelty detection algorithms, OneClassSVM, Local Outlier Factor (LOF), and KNN_average, were considered for risk-based adaptive user authentication and showed promising results. These algorithms outperformed popular machine learning classification algorithms, such as k-NN, DT, SVM, and NB, each of which demonstrated overfitting (accuracy: 10,000). In particular, KNN_average was accurate in almost all cases (99%), followed by LOF and OneClassSVM (97% and 95%, respectively) [48]. In terms of precision, recall, and F1-score evaluation metrics, the KNN_average algorithm demonstrated a slightly better performance compared to the OneClassSVM and LOF algorithms [48].

##### Papaioannou et al.

**Figure 10:**
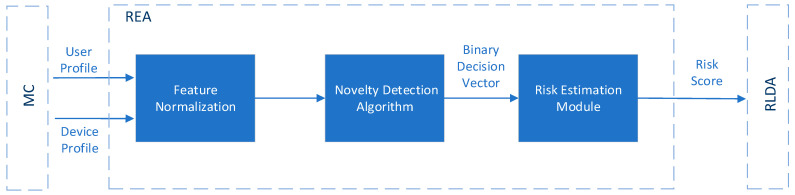
An overview of the Risk Estimation Agent (REA) proposed in [60].

**Figure 11:**
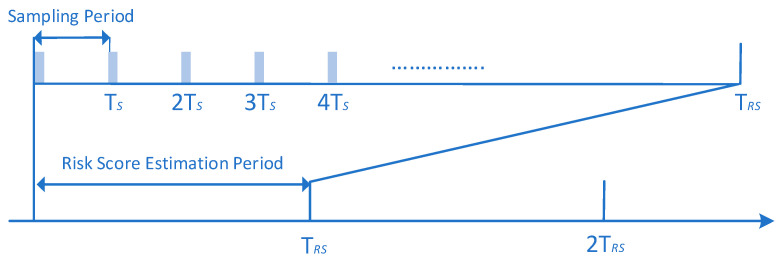
Illustration of the sampling period *Ts* and the Risk Score (RS) estimation period *T_RS_*.

Similar to the classification algorithms, the main idea is to create a formula based on which the output of one or more novelty detection algorithms will be combined to give an overall real-time risk score associated with an action or event. For instance, the authors in [60] proposed the design of a Risk Estimation Agent (REA), as depicted in Figure 10, which takes as inputs behavioral and contextual data of the user and their device (i.e., user profile and device profile), respectively, and as its outputs a real time risk score using novelty detection algorithms. More specifically, firstly, it is intended that the proposed REA component will perform data normalization to the input data (i.e., user profile and device profile) to ensure that features of the input data with substantially large values do not outweigh features with smaller values. The normalization process of the data (i.e., Feature Normalization) occurs during every sampling period Ts [60]. During this time, the Monitoring Component (MC), as depicted in Figure 10, updates both the user profile and device profile, which are referred to as input data, and sends them to the REA component. Afterward, the REA component employs the most efficient novelty detection algorithm, selected from a set of algorithms, on the normalized input data to determine if each entry into the normalized data is legitimate or malicious. Specifically, for each entry, the algorithm will output either 0 for a legitimate user or 1 for a malicious user, resulting in a binary vector of length equal to the number of normalized entries. This vector is then fed to the Risk Estimation Module, as depicted in Figure 10, which calculates the risk score, in a continuous mode, for a given period of time T_RS_. An illustration of the periods Ts and T_RS_ is shown in Figure 11.

Denoting the output (binary) vector of the novelty detection algorithms as yϵRm x 1, the risk score (i.e, $P_{0}\left(k\right)\epsilon \;\left[0,1\right]$) in a period k (e.g., *T_RS_*, *T_2RS_*) can be calculated as follows [7,60,61,62,63]

(14) $$P_{0}\left(k\right)=\frac{\sum_{i=1}^{m}y_{i}}{m}A$$

where, *A* denotes the accuracy of the novelty detection algorithms. *A* is defined as follows [60]:

(15) $$A=\frac{TP+TN}{TP+TN+FP+FN}$$

where, as defined in all machine learning algorithms, the following terms are used to describe the accuracy *A* of a model:

- True Positive (*TP*) refers to the number of positive instances (malicious users) that are correctly classified.
- True Negative (*TN*) refers to the number of negative instances (legitimate users) that are correctly classified.
- False Positive (*FP*) refers to the number of negative instances (legitimate users) that are mistakenly classified as positive (malicious users).
- False Negative (*FN*) refers to the number of positive instances (malicious users) that are mistakenly classified as negative (legitimate users).

Afterward, the calculated risk score will be sent to the Risk Level Decision Agent (RLDA) component, as illustrated in Figure 10, for comparison against the risk level thresholds stored in RLDA. The RLDA will then determine if the estimated risk score is low, medium, or high [60]. Although the authors showed that the novelty detection algorithms that they are going to use demonstrate promising results, their study lacks performance evaluation results for the whole REA component. The run-time and the computational complexity of the whole REA component are of the utmost importance as they will run on a smartphone device.

#### 3.2.4. Bayesian Networks

According to NIST publication NISTIR 8286A [32], although using expert judgement to estimate risk parameters brings significant value in risk assessments, the results of a risk assessment may be more objective and accurate when they are based on information known from prior events. Towards this direction, the deployment of Bayesian networks has caught the attention of scholars [64] as Bayesian analysis includes methods for considering conditional probability, namely the application of a distribution model and a set of known prior data to help estimate the probability of a future outcome [32].

In particular, the use of prior knowledge, obtained from internal observations and experiences from similar organizations, can significantly enhance the precision and reliability of predictions, such as determining the probability of a significant event happening or calculating the impact of that uncertainty on an enterprise’s objectives [32]. Similar techniques can also be applied to estimate the probability of multiple conditions occurring simultaneously (joint probability) or to calculate the probability of a particular outcome in light of other external factors (marginal probability) [32].

##### Luo et al.

Luo et al. [65] proposed a Bayesian network intrusion intent analysis method based on a Bayesian attack graph. Attack graphs provide powerful frameworks for risk assessments by analyzing the network topology and vulnerabilities and then creating a solid representation of the attack paths that an attacker might follow to compromise network resources. As can be observed, there is always uncertainty regarding attacker’s behavior, and thus Bayesian networks become suitable approaches to model attack graphs for static and/or dynamic risk assessments. Risk assessments, performed based on dynamic attach graphs have caught the attention of scholarship as they account for evidence of compromise at run-time, compared to risk assessments based on static attach graphs that considered the security posture at rest. As such, risk assessments based on dynamic attach graphs have shown themselves to be more efficient in helping system administrators to dynamically react against potential threats [64,65]. In their paper, Luo et al. [65] proposed a Bayesian attack graph model in order to estimate the probabilities of an attacker compromising several networks; resources. To do so, firstly, they calculated the probability of atomic attack using three evaluation indicators, namely vulnerability probability $P\left(v_{i}\right)$, attack cost $cost\left(A_{j}\right)$ and benefit $benefit\left(A_{j}\right)$:

(16) $$P\left(A_{j}\right)=\min\left(\frac{P\left(v_{i}\right)\times benefit\left(A_{j}\right)}{cost\left(A_{j}\right)},\;1\right)$$

where

(17) $$P\left(v_{i}\right)=\frac{8.22\times AV\times AC\times PR\times UI}{10}\times 100\%$$

Luo et al. [65] made use of parameters that were specified in the Common Vulnerability Scoring System (CVSS) provided by the national vulnerability database (NVD) of the US for quantification. CVSS provides comprehensive scoring parameters, an open scoring framework, a combination of dynamic assessment and vulnerability dependencies between attribute nodes, and quantification of vulnerability utilization [65]. According to the CVSS quantification standard, Luo et al. [65] used the following four indexes to quantify the vulnerability utilization probability $P\left(v_{i}\right)$, for a given vulnerability $v_{i}$: (i) Access Vector (AV), (ii) Access Complexity (AC), (iii) Privileges Required (PR) and (iv) User Interaction (UI). The authors leveraged these data to make the results of their measurement more precise and to eliminate the potential for bias due to a lack of measurement indicators [65]. The CVSS quantification standard provides comprehensive coverage of vulnerability value measurement, which the authors utilized to create a classification of impact indicators. By assigning a lower score to indicators with limited impact and a higher score to indicators with greater impact, they were able to shed light on the level of influence each indicator has on network security. This method is illustrated in Table 3, which displays the specific scores assigned to each indicator.

Accordingly, and based on the CVSS, Luo et al. [65] considered that the attack cost might be quantified using four indexes: (i) Shellcode Information (SI), (ii) Shellcode Platform (SP), (iii) Operation Requirement (OR), and (iv) Information Requirement (IR), as follows:

(18) $$cost\left(A_{i}\right)=1-\left(1-SI\right)*\left(1-SP\right)*\left(1-OR\right)*\left(1-IR\right)$$

The specific scores for SI, SP, OR, and IR are shown in Table 4, while the specific values of $benefit\left(A_{j}\right)$ are given in Table 5.

Afterwards, the authors calculated the static reachability probability, which represents the probability of each attribute node being accessible in the static network. This is determined by considering the joint conditional probability of the current node and its parent node. That is, for $S_{j}\;\in \;S_{transition}\;\cup S_{target}$, the calculation formula of the node $S_{j}$ static reachability probability is the following:

(19) $$P_{1}\left(S_{j}\right)=\overset{n}{\underset{j=1}{\prod }}P\left(S_{j}\left|P\left(S_{j}\right)\right.\right)$$

Finally, to compare the attack probability among various paths, the authors computed the total reachability probability of a path as the product of the reachability probability of each node in that path. The calculation equation for the total reachability probability of *AP_i_* is:

(20) $$P\left(AP_{i}\right)=\prod P\left(S_{i}\right),\;S_{i}\in AP_{i}\;$$

Based on their experiments, the proposed study showed efficient results compared to other similar works in the literature. The authors commented that this is because they considered more evaluation indicators, and their data were derived from CVSS. Furthermore, in addition to the value of the vulnerability, this study also considered the cost and benefit of using the attack to obtain a more accurate vulnerability assessment probability, which is more precise in the event of an actual network attack.

The authors in [64] emphasized the superiority of Dynamic Bayesian Networks (DBNs) compared to basic Bayesian Networks (BNs) in the area of risk analysis due to DBNs’ ability to model probabilistic data with consideration of temporal dependencies over time. Unlike basic BNs, which can only show relationships between variables at a specific time or for a set period, DBNs are capable of handling time-dependent risk assessments. For instance, when used in risk-based user authentication, they can demonstrate changes and relationships over time between a smartphone device’s current, past, and future states.

### 3.3. Fuzzy Logic

In the literature, there are many studies in risk assessment that apply Fuzzy Logic with a focus on predicting forest fires, reducing risk in energy management projects, and detecting anomalies in systems, but there are very few that focus on risk-based authentication for mobile devices. The purpose of this section is to investigate how fuzzy logic is utilized in other fields for risk estimation and to assess its potential for use in risk-based authentication for mobile devices. Fuzzy logic models incorporate human knowledge into usable algorithms and use two main types of sets: (i) classic or crisp sets and (ii) fuzzy sets, which can be defined by different membership functions. For instance, a classic set can be defined by a membership function as follows:

(21) $$\mu_{s}\left(X\right)=\left\{\begin{array}{c} 1\;if\;X\in \;S\\ 0\;if\;X\notin S \end{array}\right.$$

In particular, Equation (21) specifies the degree of membership of a sample point to a crisp set *S*. For the crisp sets, a function of this type is also known as characteristic function. On the other hand, in fuzzy sets which can be used to provide rational and sensible clustering, there is likewise a degree of membership $\mu_{s}\left(X\right)\in \left[0,1\right]$. The main idea is that every calculated risk belongs simultaneously to all different risk clusters (i.e., low, medium, and high risk) with a different degree of membership. As such, the characteristic cluster for each prefecture is the one with the greatest value of the membership function $\mu_{s}\left(X\right)\in \left[0,1\right]$ [45,66]. To generate different cases of Degrees of Membership depending on the application and the problem that we intend to solve, there are several membership functions that might be applied, with dissimilar shapes such as trapezoidal or triangular membership functions [67].

A trapezoidal membership function comprises a special case of the following expression [68]:

(22) $$\mu_{s}\left(X\right)=\left\{\begin{array}{c} 0\;if\;X<a\\ \frac{X-a}{m-a}if\;X\in \left[a,m\right]\\ 1\;if\;X\in \left[m,n\right]\\ \frac{b-X}{\left(b-n\right)}ifX\in \left[n,b\right]\\ 0\;if\;X>b \end{array}\right.$$

**Figure 12:**
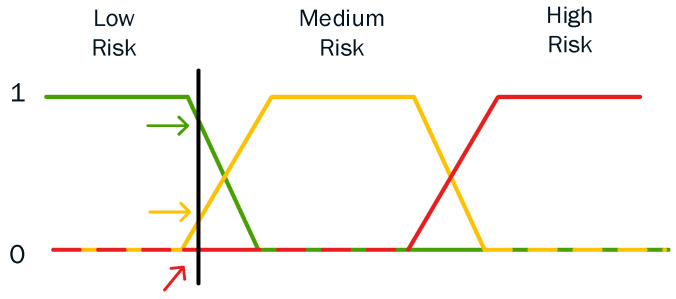
An example of the trapezoidal membership function.

An example of a trapezoidal membership function is given in Figure 12. For example, in Figure 12, three different trapezoidal functions are illustrated, representing three different expressions of low-, medium- and high-risk score, respectively. As such, every point has three “truth values”— one for each of the three functions. The vertical black line in the image represents a particular estimated risk at the current moment and the three arrows (truth values) represent the values of the three different functions for that particular point. Since the red arrow (i.e., high risk) points to zero, this risk score may be interpreted as “not high”; i.e., this risk score has zero membership in the fuzzy set “high score”. The orange arrow (pointing at virtually 0.2) may describe it as “slightly medium” and the green arrow (pointing at virtually 0.8) “fairly low”. Therefore, this risk score has 0.2 membership in the fuzzy set “medium score” and 0.8 membership in the fuzzy set “low risk”. As a result, we consider the estimated risk score to be low. The degree of membership assigned for each fuzzy set is the result of fuzzification (i.e., the process of assigning the numerical input of a system to fuzzy sets with some degree of membership).

In some cases, there is a need for a semi-form of the trapezoidal membership function which is defined as [69]:

(23) $$\mu_{s}\left(X\right)=\left\{\begin{array}{c} 0\;if\;X<a\\ \frac{X-a}{m-a}if\;X\in \left[a,m\right]\\ 1\;if\;X\in \left[m,n\right] \end{array}\right.$$

A triangular membership function comprises a special case of the following expression in Equation (24), while the semi-triangular membership function is given in Equation (25) [68]:

(24) $$\mu_{s}\left(X\right)=\left\{\begin{array}{c} 0\;if\;X<a\\ \frac{X-a}{c-a}if\;X\in \left[a,c\right]\\ \frac{b-X}{\left(b-c\right)}ifX\in \left[c,b\right]\\ 0\;if\;X>b \end{array}\right.$$

(25) $$\mu_{s}\left(\text{X}\right)=\left\{\begin{array}{c} 0\;if\;X<a\\ \frac{X-a}{c-a}if\;X\in \left[a,c\right] \end{array}\right.$$

**Figure 13:**
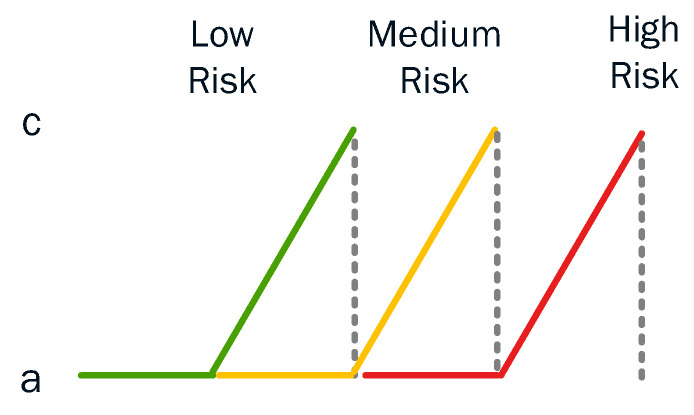
An example of the semi-triangular membership function.

In the application of forest fire prediction, Iliadis [69] states that, in practice, the triangular membership function can be used only in the semi-form and that this is because, in reality, there could not exist areas with more burned hectares (ha) than c and with a lower degree of membership to the fuzzy set ‘forest fire risky area’. This is because ‘c’ is actually the peak of the burned areas in Greece (i.e., the center of the cluster with the highest risk) and no other higher values than this can be considered. According to the results in [69], the triangular and semi-triangular (an example of which is depicted in Figure 13) membership functions performed better than the trapezoidal and semi-trapezoidal membership functions. This was expected as the triangular and semi-triangular membership functions identify clearer distinctions between the areas of highest risk. This is a significant advantage compared to the performance of the trapezoidal and semi-trapezoidal membership functions.

Membership functions are usually defined as triangle- (i.e., triangular, or semi-triangular) or trapezoid-shaped (i.e., trapezoidal or semi-trapezoidal) curves. As such as, each value of the membership functions will have a slope where the value is increasing and/or decreasing, and a peak where the value is equal to 1 (which can have a length of 0 or greater) [70]. Nevertheless, they can also be defined using a sigmoid function [71]. One commonly used sigmoid function is the standard logistic function defined as:

(26) $$S\left(x\right)=\frac{1}{1+e^{-x}}$$

The standard logistic function presents the following symmetry property:

(27) $$S\left(x\right)+S\left(-x\right)=1$$

From this, given the fact that we have 3 variables x, y, and z (peak values for low, medium and high risk, respectively), it follows that:

(28) $$\left(S\left(x\right)+S\left(-x\right)\right)\left(S\left(y\right)+S\left(-y\right)\right)\left(S\left(z\right)+S\left(-z\right)\right)=1$$

Shang et al. [72] stated that fuzzy logic models can work as a complement to probability models, assessing risks in cases where there are insufficient data and incomplete knowledge. According to them, fuzzy logic models are able to provide a framework where human reasoning and imprecise data can contribute to efficient risk analysis [72] in various environments including risk-based user authentication [48,73].

#### 3.3.1. Haslum et al.

Haslum et al. [74] proposed an online risk assessment for distributed intrusion prediction and prevention systems based on fuzzy logic. In particular, for the three different risk levels (i.e., low, medium, high), the authors proposed trapezoidal membership functions to define the “Low” and “High” values and a triangular membership function to define “Medium” value as follows:

(29) $$\;\;\mu_{L_{3}}\left(x\right)=trap\left(x,-0.4,-0.1,\;0.1,\;0.4\right)\;\mu_{M_{3}}\left(x\right)=triang\left(x,0.2,\;0.5,\;0.8\right)\;\;\mu_{H_{3}}\left(x\right)=trap\left(x,0.6,\;0.9,\;1.0,\;1.4\right)$$

Furthermore, Haslum et al. [74] used a Hidden Markov Model (HMM) that captures the interaction between the attacker and the network to model and predict the next step of a potential attacker and evaluate their proposed fuzzy online risk assessment model. On top of that, the interaction between various distributed intrusion detection systems and the efficient integration of the various output are also achieved using an HMM. The main novelty of their study is the design and development of Fuzzy Logic Controllers (one in every IDS) to estimate the numerous risk(s) that are dependent on several other variables based on the inputs from HMM modules and the DIDS agents. To establish the if-then fuzzy rules for their proposed fuzzy logic model, the authors considered interviews with security experts and network administrators. Nevertheless, their results demonstrate that the proposed online risk assessment for distributed intrusion prediction and prevention systems based on fuzzy logic is very practical and highly effective in practice when it comes to protection assets which are highly at risk of misuse and/or attacks [74]. In addition, their implementation is very simple, while the developed system is easy to interpret [74]. The authors are planning to design and develop adaptive fuzzy inference systems when some preliminary data or knowledge related to network risk is available. On top of that, they are aiming to examine the use of different fuzzy inference methods for several applications and use cases [74].

#### 3.3.2. Gusmão et al.

**Figure 14:**
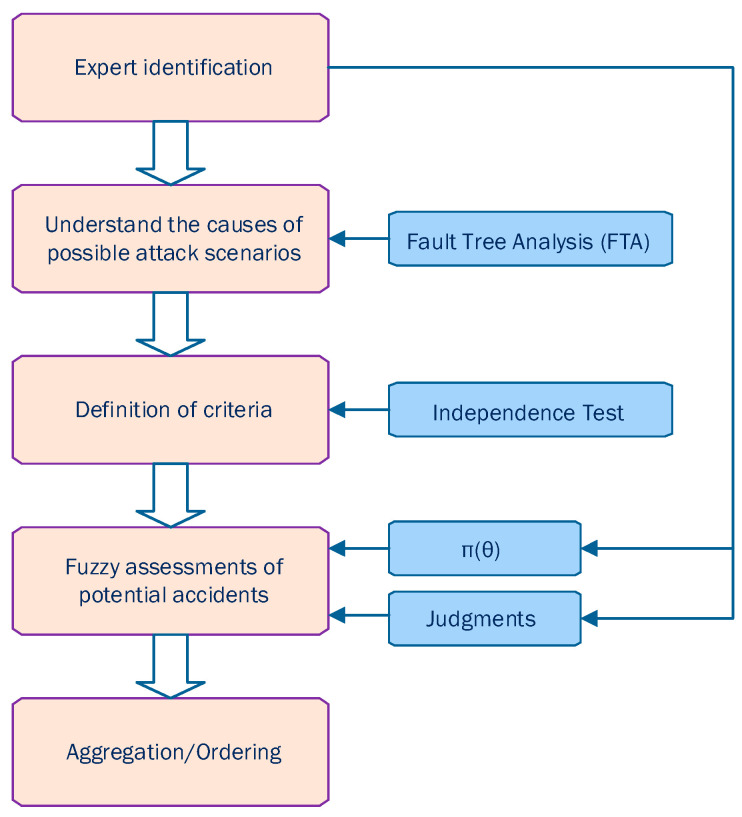
The five phases of the proposed cybersecurity risk analysis model using fuzzy logic and fault tree analysis [75].

Last but not least, Gusmão et al. [75] proposed a cybersecurity risk analysis model using fuzzy logic and fault tree analysis. Their proposed cybersecurity risk analysis model includes five phases as depicted in Figure 14: (i) expert identification, (ii) understanding the causes of possible attack scenarios, (iii) definition of criteria, (iv) fuzzy assessment and finally, and (v) aggregation and ordering. Its main aim is to provide practical support for controlling and evaluating cybersecurity attacks, including, for example, evaluations of the consequences of a cyberattack and evaluations of the effects of a potential cyberattack, in terms of some define criteria.

Further, Gusmão et al. [75] utilized Fault Tree Analysis (FTA) to detect scenarios that lead to hazards. The method they followed for conducting FTA is described in Table 6. This structured analysis helped to identify key aspects and assess the vulnerability of cybersecurity as well as the potential impact of cyberattacks. To demonstrate the usefulness of the proposed model, Gusmão et al. [75] developed an example using three different evaluation alternatives: a website, ERP, and e-commerce. They evaluated the consequences of data dissemination, data modification, data loss or destruction, and service interruption in terms of financial costs and time to restoration. The results of the model’s application show its usefulness and suggest that e-commerce may be more vulnerable to cybersecurity attacks compared to other websites or ERP, partially due to frequent operator access, credit transactions, and user authentication issues.

### 3.4. Non-Graph-Based

#### Gehani et al.

**Figure 15:**
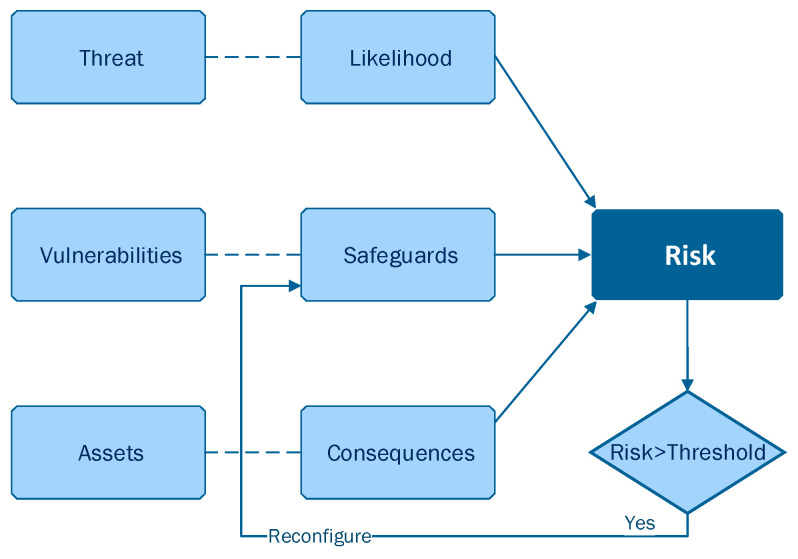
RheoStat: Real-Time Risk Management [76].

Gehani et al. [76] proposed a framework for systematic fine-grained response, which is to be achieved by dynamically controlling the host’s exposure to perceived threats. In particular, their proposed model, namely RheoStat, calculates a real-time risk score representing the risk faced by a host based on the popular qualitative risk assessment [77,78]. As depicted in Figure 15, Gehani et al. [76] suggested that risk can be computed as a function of: (i) the threats, (ii) their likelihood, (iii) the vulnerabilities, (iv) the safeguards, (v) the assets and (vi) the consequences. Afterwards, they quantified each of these elements to form a final equation for the overall real-time risk score.

First of all, the authors defined the threat as a possible attack to any application or system software that is running on the host and they considered it to be characterized by an intrusion detection signature, denoted as $T=\left\{t_{1},t_{2},\ldots \right\}$, where $t_{a}\in T$ represents a host-based intrusion detection signature. Thus, $t_{a}$ will comprise an ordered set of events $S\left(t_{a}\right)=\left\{s_{1},s_{2},\ldots \right\}$.

Then, the authors defined likelihood as the hypothetical probability of a threat to actually occur and they considered a function μ in order to compute the likelihood of threat $t_{a}$ occurring. Given the fact that μ depends on the history of systems events which are relevant to this particular intrusion detection signature, if all events that have occurred are denoted as $E=\left\{e_{1},e_{2},\ldots \right\}$, then:

(30) $$T\left(t_{a}\right)=\mu (t_{a},E\overset{≺}{\cap }S\left(t_{a}\right))$$

where $\overset{≺}{\cap }$ yields the set of all events that occur in the same order in each input set.

Next, the authors defined an asset as an item that has value to the system, and they considered assets as data stored in the system, and denoted as $O=\left\{o_{1},o_{2},\ldots \right\}$ the set of assets where $o_{\beta }\in O$. Then, the authors considered a set of objects $A\left(t_{a}\right)\subseteq O$ to be associated with each threat $t_{a}$, and thus only objects $o_{\beta }\in A\left(t_{a}\right)$ could be harmed if threat $t_{a}$ is successfully introduced.

Afterwards, the authors defined a consequence as a type of harm that an asset (i.e., data) might suffer if a threat actually occurs and they considered there to be three types of consequences, namely loss of integrity $i(o_{\beta })$, confidentiality $c(o_{\beta })$, and availability $a(o_{\beta })$. If the attack does not affect a certain consequence, then the respective value for this type of consequence will be 0. Nevertheless, for $o_{\beta }\in A\left(t_{a}\right)$ to hold, all three values associated with a single object cannot be 0 at the same time. Hence, the consequence of a threat $t_{a}$ is:

(31) $$C\left(t_{a}\right)=\underset{o_{\beta }\in A\left(t_{a}\right)}{\sum }i(o_{\beta })+c(o_{\beta })+a(o_{\beta })$$

In the following, the authors defined a vulnerability as a weakness in the system that might result from an error in its design, implementation, or configuration, and they considered the set of vulnerabilities in their system $W=\left\{w_{1},w_{2},\ldots \right\}$. Hence, $W\left(t_{a}\right)\subseteq W\;$represents the set of system weaknesses exploited by $t_{a}$.

The authors then defined a safeguard as a mechanism that controls the exposure of the system’s assets. In an operating system, the set of permission checks $P=\left\{p_{1},p_{2},\ldots \right\}$, which is performed by the reference monitor and serves as a safeguard. As the reference monitor mediates access to all objects, limiting a vulnerability’s exposure can be achieved by denying the relevant permissions. The set $P_{w_{\gamma }}\subseteq P$ includes all the permissions requested during the exploitation of vulnerability $w_{\gamma }$. In a conventional reference monitor, the static configuration either grants or denies access to a permission $p_{⋋}$, represented by $v\left(p_{⋋}\right)$, with a value of either 0 or 1. The active reference monitor can reduce the exposure of a statically granted permission to $v^{\prime }\left(p_{⋋}\right)$, a value in the range [0, 1]. This reflects the nuance that results from evaluating predicates as auxiliary safeguards. Thus, if all auxiliary safeguards are utilized, the total exposure to a threat $t_{a}$ is:

(32) $$V\left(t_{a}\right)=\underset{p_{⋋}\in \hat{P}\left(t_{a}\right)}{\sum }\frac{v\left(p_{⋋}\right)\times v^{\prime }\left(p_{⋋}\right)}{\left|\hat{P}\left(t_{a}\right)\right|}$$

where:

(33) $$\hat{P}\left(t_{a}\right)=\underset{w_{\gamma }\in W\left(t_{a}\right)}{\cup }P\left(w_{\gamma }\right)$$

Finally, the risk to the host is the accumulation of the risks caused by all the threats it faces. The risk from a single threat is calculated by multiplying the likelihood of the attack happening, the system’s exposure to the attack, and the cost of the attack’s consequences [76,79]. Hence, the total risk faced by the system is:

(34) $$R=\underset{t_{a}\in T\;}{\sum }T\left(t_{a}\right)\times V\left(t_{a}\right)\times C\left(t_{a}\right)$$

Gehani et al. [76] state that, in order to effectively manage the risk posed to the system, its level must be continuously monitored at all times. If the risk surpasses the tolerance level of the host, the system’s security needs to be strengthened. Conversely, if the risk decreases, the restrictions can be loosened in order to enhance performance and usability. The risk to a system can be decreased by reducing the exposure of vulnerabilities through the implementation of auxiliary safeguards before granting a permission. Additionally, if the threat decreases, the restrictive permission checks can be relaxed.

Gehani et al. [76] developed and tested a prototype in Sun’s Java Runtime Environment (version 1.4.2), running on Redhat Linux 9 (with kernel 2.4.20). The utility of their proposed framework is illustrated with a set of attack scenarios in which the risk is managed in real-time and results in the attacks being contained [76]. This is very promising for estimating the real-time risk score when deploying risk-based user authentication on smartphone. The timely and accurate risk estimation (i.e., computation of an accurate risk score of an action or event) plays a key role in risk-based continuous user authentication as it might impact its overall usability and security.

### 3.5. Monte Carlo Simulation

Monte Carlo Simulation (MCS) is a robust simulation model for risk assessment that calculates the risk scores of numerous possible scenarios, taking into account the many random variables that play crucial roles in cybersecurity risks [32,45]. MCS is practical and can be easily performed on a standard personal computer [30]. The model involves using a conventional computer to generate a large number of scenarios based on randomly sampled probabilities as conventional inputs. For each scenario, MCS generates specific values for each unknown variable. These values are then used in a formula to calculate an output for each scenario. This process is typically repeated for thousands of scenarios.

Consequently, MCS is able to generate a comprehensive probability distribution for risk scores, providing accurate results. According to NISTIR 8286A [32], the simulation results can be easily interpreted through visualization on a graph, such as a histogram. The MCS method reduces subjectivity by simulating the random behavior of a system through a set of experiments, as opposed to relying on expert judgement [32,80]. While it requires high computing power, the growing availability of high-speed computers has made the MCS increasingly popular. The main advantage of the MCS is that it is able to work with large and complex systems. Moreover, MCS can manage the probabilistic behavior of several inputs to the system, which in the analytical technique are supposed to be constant values [80]. This is very promising for the risk estimation process in risk-based continuous user authentication that relies on behavioral biometrics for calculating the risk score, as the overall real-time risk score takes into consideration large and complex input data (i.e., behavioral biometrics, such as free-text keystroke sequences).

## 4. Conclusions

Risk-based continuous user authentication, relying on behavioral biometrics, has been shown to have the potential to further improve mobile authentication security without sacrificing usability (i.e., security and usability are often thought of as being contradictory) [12,21,22,23]. More specifically, risk-based continuous user authentication mechanisms manage to continuously verify the user’s identity during their entire interaction with the smartphone device based on a real-time risk score. In particular, when an abnormality on a set of attributes regarding user’s behavior has been detected (i.e., event-driven approach), a real-time risk score is estimated, and if this risk score is above an acceptable threshold (i.e., high risk score), re-authentication will be triggered. It is clear that accurate risk estimation (i.e., computation of an accurate risk score of an action or event) plays a key role in risk-based continuous user authentication as it might impact its overall usability and security [27,28].

In principle, the different approaches, proposed over the years, for risk estimation can be qualitative or quantitative [29]. Qualitative risk estimation approaches have been widely used in several areas until recently [81]. However, despite their apparent rationality, current qualitative approaches rely heavily on expert intuition, resulting in subjective risk assessments. This makes them unsuitable for practical security solutions in real-world scenarios [30,31]. On the other hand, quantitative risk estimation approaches have emerged as a new tendency, addressing the subjective nature of qualitative risk estimation approaches and improving accuracy [32]. Nevertheless, so far, there is a lack of suitable quantitative risk estimation approaches for risk-based continuous user authentication on smartphones. Therefore, in the context of this study, we present an extensive overview of existing quantitative risk estimation approaches (QREA) found in the literature not only for risk-based user authentication on mobile devices, but also for other security applications such as user authentication in web/cloud services, intrusion detection systems, etc., that could be possibly adopted in risk-based continuous user authentication solutions for smartphones. The target of this study was to provide a foundation for organizing research efforts toward the design and development of proper quantitative risk estimation approaches for risk-based continuous user authentication solutions for smartphones. The reviewed quantitative risk estimation approaches have been divided into the following five main categories: (i) probabilistic approaches, (ii) machine learning-based approaches, (iii) fuzzy logic models, (iv) non-graph-based model, and (v) Monte Carlo simulation models. Table 7 summarizes our findings.

As future research, we plan to take into consideration the outcome of this study, in terms of the strengths and weaknesses of the examined QREAs, and design and develop reliable and efficient quantitative risk estimation approaches for risk-based continuous user authentication solutions for smartphones. Finally, the developed risk estimation approaches will be evaluated based on their computational cost, communication overhead, and storage overhead when integrated into a risk-based continuous user authentication mechanism running on a smartphone device.

## Figures and Tables

**Table 1:** User parameter weights [49].

User Parameter	User Parameter Weight
Browser version	1
OS version	2
Login time	3
IP address	4
Device type	5
No. of failed attempts	6
Geolocation	7
Time zone	8

**Table 2:** Additional authentication methods required based on the identified risk levels [49].

SVM and NB Classifiers Output (i.e., Probabilities P)	One-Class SVM Output (i.e., Risk Score S)	Risk Level	Action Required (i.e., Authentication Method)
50≤P≤60	1≤S≤6	1	Security questions
60<P≤75	7≤P≤18	2	OTP token
75<P≤90	19≤P≤29	3	Graphical password
90<P≤100	30≤P≤36	4	Digital signature

**Table 3:** CVSS indicator scores used by Luo et al. [65].

Indexes	Measurements	Score
AV	Network (N)	0.85
	Adjacent (A)	0.62
	Local (L)	0.55
	Physical (P)	0.20
AC	Low (L)	0.77
	High (H)	0.44
PR	None (N)	0.85
	Low (L)	0.62
	High (H)	0.07
UI	None (N)	0.85
	Required (R)	0.62

**Table 4:** Attack cost index score used by Luo et al. [65].

Cost	Measurements	Score
SI	Complete/Function/Null	0.1/0.3/0.7
SP	Common/Special/Particular	0.15/0.35/0.6
OR	Tool/Script/Manual/Corporation	0.1/0.25/0.45/0.7
IR	Null/Regular/Configuration/Critical	0/0.2/0.55/0.8

**Table 5:** Attack benefit index score used by Luo et al. [65].

Measurements	Score
Information leakage	0.3–0.55
Remote register	0.55–0.7
Authentication bypass	0.7–0.8
Limited access	0.85–0.95
Root access	1.0

**Table 6:** Procedure for FTA [75].

Step	Definition
Step 1	Define the system of interest, regarding the cyberattacks and other events as initial conditional causes of failure in the security system.
Step 2	Define the top event for the analysis and specify the problem of interest that the analysis will address.
Step 3	Define the treetop structure. Determine the events and conditions (i.e., intermediate events) that lead most directly to the top event, which in this case can be faulty network and fault IS.
Step 4	Explore each branch in successive levels of detail. Determine the events and conditions that lead most directly to each intermediate event.

**Table 7:** Taxonomy of Quantitative Risk Estimation Approaches (QREAs).

Reference	Used Technique	Observations	Platform	
			Smartphone	Other
Freeman et al. [33]	Probabilistic	Proposed to estimate a risk score for a login attempt in a web service to classify the login attempt into normal or suspicious, strengthening this way password-based authentication. Freeman et al. approach showed to be the most suitable for categorical data (i.e., IP address and useragent) for scalable and practical RBA solutions that can be used in large-scale online services.		x
Arnes et al. [35]	Probabilistic; Hidden Markov Model	Proposed to estimate a real-time risk score approach for intrusion detection systems based on observations from network sensors, showing promising results. It can be the basis for automated response IDSs		x
Chen et al. [34]	Probabilistic; Hidden Markov Model	Proposed to evaluate system risk based on software behavior using HMMs. The approach has demonstrated credible results for risk assessment in a quantitative manner and can be utilized in the actual risk assessment process for various applications.		x
Mu et al. [38]	Probabilistic; D–S Evidence	Proposed for online risk assessment of intrusion scenarios. The deployment of the proposed risk assessment model enables IDAM&IRS to tolerate IDS false positive alerts setting the foundation for effective intrusion response decision-making.		x
Acien et al. [40]	ML-based; Siamese Neural Networks	Proposed for keystroke dynamics behavioral biometric to effectively authenticate 100 K users typing free-text, when the amount of data per user is very limited: a common scenario in free-text keystroke authentication. Demonstrated efficient performance in terms of EER and computational time. Demonstrated potential use of SNN in risk-based user authentication based on behavioral biometrics.	x	
Misbahuddin et al. [49]	ML-based; Classification Algorithms	Proposed to determine the risk score every time the user sings-in in a web application. Demonstrated promising results in terms of effectively classifying the user as genuine or suspicious while ensuring usability. The authors deployed 3 classifiers, namely SVM, one-class SVM, and NB, and suggested that one-class SVM classifier is more efficient in real-world applications where there is not enough data for both classes (i.e., genuine and suspicious).		x
Papaioannou et al. [48]	ML-based; Novelty Detection Algorithms	Proposed to estimate a real-time risk score in risk-based user authentication for smartphone devices. The deployed novelty detection algorithms demonstrated high-performance evaluation results. However, performance evaluation results of the whole REA component were not provided.	x	
Luo, Z. [65]	ML-based; Dynamic Bayesian Attach Graph	Efficient results compared to similar works of research in the literature. The used data were derived from CVSS standard. In addition to the value of the vulnerability, it also considered the cost and benefit of the attack to obtain a more accurate vulnerability assessment probability, which is more precise given an actual network attack. Ideal for handling time-dependent risk assessments, for instance when they are used in risk-based user authentication, they are able to present changes over time and relationships between a smartphone device’s current, past or future states.		x
Iliadis L.S. [69]	Fuzzy Logic	Proposed for forest fire prediction. Suggested that the triangular and semi-triangular membership functions performed better than the trapezoidal and semi-trapezoidal membership functions.		x
Shang et al. [72]	Fuzzy Logic	Suggested that fuzzy logic models can serve as a complementary approach to probability models, particularly in cases where data are limited and knowledge is incomplete. Fuzzy logic models provide a framework that allows human reasoning and imprecise data to contribute to efficient risk analysis in various applications, including risk-based user authentication.		x
Haslum et al. [74]	Fuzzy Logic	Proposed for distributed intrusion prediction and prevention systems. Demonstrated to be very practical and highly effective in practice when it comes to protect assets which are highly at risk of misuse and/or attacks.		x
Gehani et al. [76]	Non-Graph-based; Addition, Multiplication & Division	Proposed for achieving a systematic, fine-grained response by dynamically managing a host’s exposure to perceived threats. The approach has been shown to be highly effective in a series of attack scenarios, where risk is managed in real-time, leading to the containment of attacks.		x
Goerdin et al. [80]	Monte Carlo Simulation	Capable of handling intricate and substantial systems. Able to regulate the uncertain behavior of multiple inputs to the system, which are considered constant values in analytical methods.		x

## Data Availability

Not applicable.
